# Retraction: Anti-angiogenic effects of *Moringa oleifera* silver nanoparticles on endothelial cells: in vitro and ex vivo studies

**DOI:** 10.37349/etat.2025.1002343

**Published:** 2025-10-28

**Authors:** Editorial Office

The above article, published online on 27 July 2025 in *Exploration of Targeted Anti-tumor Therapy* (https://www.explorationpub.com/Journals/etat), has been retracted by agreement between the Editor-in-Chief, Nicola Normanno, and the editorial team. This retraction was prompted by issues identified with the figures in the manuscript during the journal's routine systematic self-examination.

The specific concerns uncovered are detailed below:

- Figure 4 shows an unexpected duplication between panels B and C.
- Figure 5 shows an unexpected duplication between panels B, C, and D.
- Figure 7 shows an unexpected duplication between panels “Quercetin 15.11 μg/mL, Day 3”, “MO-AgNPs 12 μg/mL, Day 8”, and “MO-AgNPs 6 μg/mL, Day 14”; “MO-AgNPs 12 μg/mL, Day 3” and “MO-AgNPs 6 μg/mL, Day 8”.

Upon identifying the issues, the journal promptly reached out to the authors, requesting a self-assessment. The authors have taken a cooperative stance by providing explanations and documents when approached for clarification, but failed to provide an adequate response to demonstrate adherence to the journal's standards. As a result, the article has been retracted with the approval of the editorial team.

All authors disagree with the retraction.

